# ABHD4 regulates adipocyte differentiation *in vitro* but does not affect adipose tissue lipid metabolism in mice

**DOI:** 10.1016/j.jlr.2023.100405

**Published:** 2023-06-22

**Authors:** Mary E. Seramur, Sandy Sink, Anderson O. Cox, Cristina M. Furdui, Chia-Chi Chuang Key

**Affiliations:** 1 Department of Internal Medicine, Section on Molecular Medicine, Wake Forest University School of Medicine, Winston Salem, NC, USA; 2 Wake Forest Baptist Comprehensive Cancer Center Proteomics and Metabolomics Shared Resource, Wake Forest University School of Medicine, Winston Salem, NC, USA

**Keywords:** Adipocyte differentiation, Alpha/beta hydrolase domain, ABHD4, Lipogenesis, Lipolysis, N-acyl ethanolamine, Oleoylethanolamide, Obesity

## Abstract

Alpha/beta hydrolase domain-containing protein 4 (ABHD4) catalyzes the deacylation of *N*-acyl phosphatidyl-ethanolamine (NAPE) and lyso-NAPE to produce glycerophospho-*N*-acyl ethanolamine (GP-NAE). Through a variety of metabolic enzymes, NAPE, lyso-NAPE, and GP-NAE are ultimately converted into NAE, a group of bioactive lipids that control many physiological processes including inflammation, cognition, food intake, and lipolysis (i.e., oleoylethanolamide or OEA). In a diet-induced obese mouse model, adipose tissue *Abhd4* gene expression positively correlated with adiposity. However, it is unknown whether *Abhd4* is a causal or a reactive gene to obesity. To fill this knowledge gap, we generated an *Abhd4* knockout (KO) 3T3-L1 pre-adipocyte. During adipogenic stimulation, *Abhd4* KO pre-adipocytes had increased adipogenesis and lipid accumulation, suggesting *Abhd4* is responding to (a reactive gene), not contributing to (not a causal gene), adiposity, and may serve as a mechanism for protecting against obesity. However, we did not observe any differences in adiposity and metabolic outcomes between whole-body *Abhd4* KO or adipocyte-specific *Abhd4* KO mice and their littermate control mice (both male and female) on chow or a high-fat diet. This might be because we found that deletion of *Abhd4* did not affect NAE such as OEA production, even though *Abhd4* was highly expressed in adipose tissue and correlated with fasting adipose OEA levels and lipolysis. These data suggest that ABHD4 regulates adipocyte differentiation in vitro but does not affect adipose tissue lipid metabolism in mice despite nutrient overload, possibly due to compensation from other NAPE and NAE metabolic enzymes.

In the United States, from 1999 through 2018, the prevalence of adult obesity increased from 30.5% to 42.4% (https://www.cdc.gov/obesity/data/adult.html). The most common chronic conditions associated with adult obesity include type 2 diabetes, cardiovascular disease, stroke, and cancer which overall cause about 1 in 5 deaths in the United States each year (1, 2). Obesity is caused by a chronic imbalance between energy intake and energy expenditure resulting in an increase in both adipocyte number (i.e., hyperplasia) and adipocyte size (i.e., hypertrophy) (3, 4). The worldwide epidemic of obesity has spurred an increased interest in understanding the molecular mechanisms regulating adipocyte hyperplasia and hypertrophy and in developing new therapeutic approaches (3, 4).

The alpha/beta hydrolase domain-containing proteins (ABHDs) play an important role in the regulation of lipid metabolism and signal transduction (5, 6). For example, ABHD5, also known as comparative gene identification-58 (CGI-58), is highly expressed in adipose tissue, and during lipolysis, ABHD5/CGI-58 coactivates adipose triglyceride lipase (ATGL) to catalyze triacylglycerol (TAG) hydrolysis, resulting in free fatty acid and glycerol release into the circulation. Mutation of ABHD5 disrupts lipolysis leading to adiposity and ectopic lipid accumulation in *Arabidopsis* (7), *C. elegans* (8), mice (9), and humans (i.e., Chanarin-Dorfman syndrome) (10).

ABHD4 is a paralog of ABHD5 (ABHD4 shares 50%–55% sequence identity with ABHD5), but it does not affect ATGL activity. In 2006, Simon and Cravatt first identified that ABDH4 is a phospholipase/lyso-phospholipase B that catalyzes the deacylation of *N*-acyl phosphatidyl-ethanolamine (NAPE) and lyso-NAPE to produce glycerophospho-*N*-acyl ethanolamine (GP-NAE) (11), an alternative pathway compared to a classical NAPE-phospholipase D (NAPE-PLD) pathway (Fig. 1A). They further described that glycerolphosphodiester phosphodiesterase 1 (GDE1) catalyzes the hydrolysis of GP-NAE to produce glycerol-3-phosphate and NAE (12) (Fig. 1A). NAE is a group of bioactive lipids including palmitoylethanolamide (PEA), stearoylethanolamide (SEA), oleoylethanolamide (OEA), linoleoylethanolamide (LEA), and arachidonoylethanolamide/anandamide (AEA) that regulate many physiological processes including pain, inflammation, anxiety, cognition, food intake, and lipolysis (13). Recently, the Ueda lab revealed two new enzymes, GDE4 (also known as glycerolphosphodiester phosphodiesterase domain-containing protein 1 or GDPD1) (14) and GDE7 (also known as GDPD3) (15), that can convert lyso-NAPE to lyso-phosphatidic acid (lyso-PA) and NAE (Fig. 1A).

**Fig. 1:**
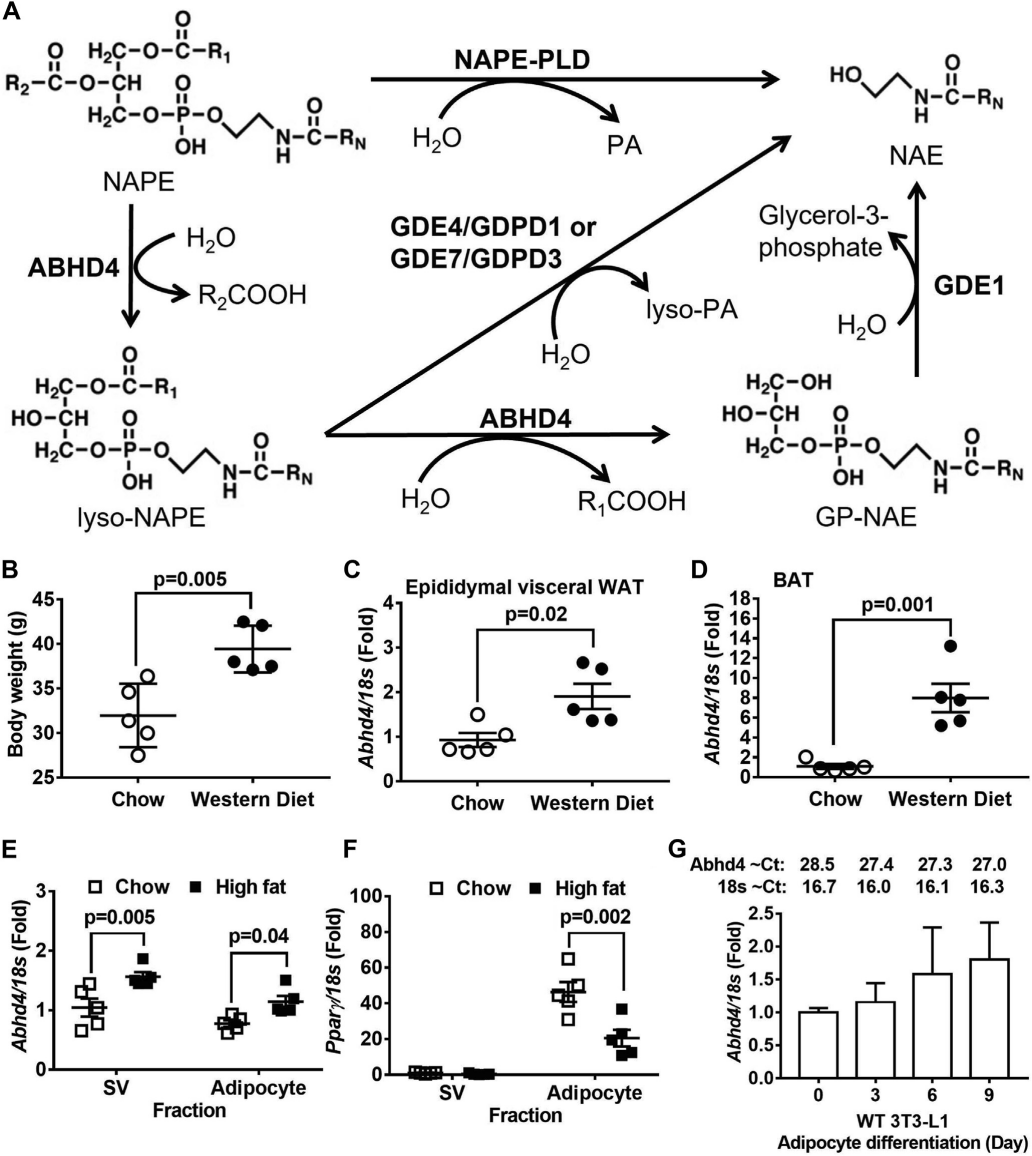
ABHD4’s role in lipid metabolism and its relationship to adiposity. A: The classical pathway to convert *N*-acyl phosphatidyl-ethanolamine (NAPE) to *N*-acyl ethanolamine (NAE) is through NAPE-phospholipase D (NAPE-PLD). Enzymes involving in an alternative pathway or NAPE-PLD independent pathway for NAE production include alpha/beta hydrolase domain-containing protein 4 (ABHD4), glycerolphosphodiester phosphodiesterase 1 (GDE1), GDE4/glycerolphosphodiester phosphodiesterase domain-containing protein 1 (GDPD1), and GDE7/GDPD3. ABHD4 catalyzes the deacylation of NAPE and lyso-NAPE to produce glycerophospho-*N*-acyl ethanolamine (GP-NAE). GDE1 catalyzes the hydrolysis of GP-NAE to produce glycerol-3-phosphate and NAE. GDE4/GDPD1 and GDE7/GDPD3 can convert lyso-NAPE to lyso-phosphatidic acid (lyso-PA) and NAE. B: Eight-week-old male C57BL/6J mice were fed chow or a Western diet for 16 weeks (n = 5/diet group) and their body weight was measured. Mice were then fasted for 24 h and their (C) epididymal white adipose tissue (WAT) and (D) interscapular brown adipose tissue (BAT) RNA was extracted and reverse-transcribed into cDNA for real-time PCR quantification of *Abhd4* normalized to *18s* (endogenous control). Results are presented as the fold change compared to chow-fed mice. B–D: Data are analyzed using a two-tailed Student's unpaired *t* test. E, F: Six-week-old male C57BL/6J mice were fed chow or a high fat diet for 12 weeks (n = 5/diet group). Adipocyte and stromal vascular (SV) cell fractions were isolated from the epididymal visceral WAT of overnight fasted mice. Both fractions’ RNA was extracted and reverse-transcribed into cDNA for real-time PCR quantification of *Abhd4* and *Pparγ* normalized to *18s* (endogenous control). Results are presented as the fold change compared to chow SV cell fraction and analyzed using a two-way ANOVA with Sidak multiple comparisons. G: Wild-type (WT) 3T3-L1 cells (n = 3/time point) were proliferated for 2 days (Day 0) and then differentiated into mature adipocytes for 3, 6, and 9 days. Cellular RNA was extracted and reverse-transcribed into cDNA for real-time PCR quantification of *Abhd4* normalized to *18s* (endogenous control). Results are presented as the fold change compared to Day 0 of WT 3T3-L1 and analyzed using a one-way ANOVA with Dunnett's multiple comparisons.

Studies have shown that deletion of *Nape-pld* in hepatocytes and adipocytes results in TAG accumulation in the liver (i.e., steatosis) (16) and adipose tissue (i.e., adiposity/obesity) (17), respectively. However, the underlying mechanisms are still unclear. In addition, our group and others have shown that GDE1 (18) and GDE7/GDPD3(20) regulate liver TAG metabolism by controlling the production of glycerol-3-phosphate and lyso-PA, respectively. Both glycerol-3-phosphate and lyso-PA are intermediate substrates of the Glycerol Phosphate Pathway for de novo TAG synthesis. A whole-body *Abhd4* knockout (KO) mouse has been generated using a standard gene targeting approach to study its role in NAPE and NAE metabolism in the central nervous system (19). However, it is still unclear whether ABHD4 is involved in NAPE and NAE metabolism in the peripheral tissues, and whether it is associated with any metabolic phenotypes in vivo. Therefore, in this study, we wanted to investigate the functional role of ABHD4 in adipocyte differentiation, adipose tissue lipid biology, and systemic metabolism. To do so, our lab has generated an *Abhd4* KO 3T3-L1 pre-adipocyte and a floxed *Abhd4* mouse for the creation of an adipocyte-specific *Abhd4* KO mouse. We also obtained a whole-body *Abhd4* KO mouse created using CRISPR gene editing from the Jackson Lab.

## Materials and Methods

### Cell culture

The 3T3-L1 pre-adipocyte cell line was purchased from ATCC (CL-173™). The *Abhd4* KO 3T3-L1 pre-adipocyte was generated using CRISPR gene editing and provided by Synthego Corporation (cells are available upon request). The guide sequence (i.e., TTATGTATCCCTCCCAAACC) was designed to target exon 3 of *Abhd4* (supplemental Fig. S1A). The KO clone was cut and had an extra nucleotide (i.e., T) insertion during the non-homologous end joining repair process, resulting in a frameshift mutation that causes premature termination of translation at a new nonsense codon, as confirmed by the Sanger sequence (supplemental Fig. S1A).

The *Abhd4* KO 3T3-L1 and its wildtype (WT) control 3T3-L1 pre-adipocytes were cultured and differentiated into adipocytes as described previously (20). Briefly, pre-adipocytes were seeded at 0.05 × 10^6^ cells per well in a 6-well culture plate (for RNA extraction, microscopic imaging, and radioisotope experiments), and 0.1 × 10^6^ cells per 60 mm culture dish (for Oil Red O staining), and 0.2 × 10^6^ cells per 100 mm culture dish (for protein collection and TAG mass analysis). Cells were cultured in Dulbecco’s Modified Eagle Medium (DMEM, Gibco) supplemented with 10% iron-fortified calf serum (Sigma-Aldrich) and 1% penicillin/streptomycin (P/S, Gibco) for 48 h until ∼90% cell confluence. Adipogenesis (Day 0) was induced by changing the medium to DMEM containing 10% fetal bovine serum (FBS; Sigma-Aldrich) plus an adipogenic cocktail (Sigma-Aldrich) including 1 μg/ml of insulin, 0.25 μM of dexamethasone, 0.5 mM of 3-isobutyl-1-methylxanthine, and 2 μM of rosiglitazone for 3 days (Day 3). Cells were then treated with 1 μg/ml of insulin only for 3 days (Day 6) and then without any adipogenic reagents for the next 3 days (Day 9). The medium was changed every 2 days.

### RNA extraction and real-time PCR

Total RNA was harvested from cells and tissues using Qiazol Lysis Reagent and isolated by following the protocol described in the RNeasy Lipid Tissue Mini Kit (Qiagen). The concentration and quality of RNA were determined using a Nanodrop (ThermoFisher Scientific) and standardized to 1 μg of RNA for cDNA synthesis. The cDNA was prepared with the OmniScript RT Kit (Qiagen) and stored at −20°C until used for Real-time PCR. Real-time PCR was performed in duplicate on the 7500 Real-Time PCR Systems using TaqMan® Fast Advanced Master Mix and TaqMan® gene expression assays (ThermoFisher Scientific) including *Abhd4* (Mm00506368), *Pparg* (Mm0040940_m1), *Srebf1* (Mm00550338_m1), *Acc1* (Mm01304257_m1), *Fasn* (Mm00662319_m1), *Fabp4* (Mm00445878_m1), *Cd36* (Mm00432403_m1), *Agpat9* (Mm04211965), *Agpat6* (Mm04211965), *Agpat1* (Mm0047900_m1), *Agpat2* (Mm00458880_m1), *Dgat1* (Mm00515643_m1), and *Dgat2* (Mm00499536_m1). Gene expression was normalized to the endogenous control gene *18S* rRNA (REF 4352655) and analyzed using the 2^ddCt^ method with 95% confidence (21).

### Lipid extraction and TAG measurement

Cells were washed with ice-cold 1X Dulbecco’s phosphate-buffered saline with no added magnesium or calcium (DPBS, Gibco) twice and lipid-extracted with hexane:isopropanol (3:2, vol:vol) overnight at room temperature. The lipid extracts were dried under a nitrogen stream at 60°C. Once dry, 1% Triton-X in chloroform was added and then dried down again. The residue was re-suspended in ddH_2_O and heated at 60°C for 1 h to yield an aqueous lipid extract for each sample. The TAG levels were measured using an enzymatic assay (Wako Diagnostics L-Type TG M) according to manufacturer instructions. After lipid extraction, cells were dissolved with 0.1 N of NaOH, and protein concentration was measured using a Pierce™ BCA Protein Assay Kit (Life Technologies) for protein normalization (22).

### Microscopic imaging and Oil red O staining

Cells were imaged on the 6-well plate at 10× magnification with a set scale of 100 μm using Nikon eclipse TE2000 inverted microscope. Another set of cells was used for Oil Red O staining. Briefly, cells were washed twice with 1X DPBS. Cells were then fixed in 10% phosphate-buffered formalin for 30 min at room temperature. After fixation, the cells were washed twice again with 1X DPBS and then treated with Oil Red O, four parts ddH_2_O with six parts Oil Red O solution (Sigma-Aldrich), for 15 min. Each well was gently washed 5 times with ddH_2_O and then imaged (22).

### Fatty acid uptake and lipogenesis

Fatty acid uptake and incorporation into lipids as well as de novo lipogenesis were determined using [^3^H-oleic acid and [^14^C]-acetic acid, respectively, following the procedure adapted from our previous study (22). Day 7 differentiated *Abhd4* KO and WT 3T3-L1 adipocytes were labeled with 0.5 μCi of [1,2–^14^C]-acetic acid (PerkinElmer) or 5 μCi of [9,10-^3^H(N)]-oleic acid (PerkinElmer) plus 0.04 mM oleic acid (Sigma-Aldrich) conjugated with 0.01 mM fatty acid free-bovine serum albumin (BSA) per ml of DMEM supplemented with 10% FBS, 1% P/S and 100 nM insulin for 0 (no radioisotopes), 30, 60 and 120 min. Following radiolabeling, cells were washed with ice-cold DPBS twice and lipid-extracted with hexane:isopropanol (3:2, vol:vol). Lipid classes from standards and cellular lipid extracts were separated by thin layer chromatography using Silica Gel plates and a solvent system containing hexane:diethyl ether:acetic acid (80:20:2, vol:vol:vol). Lipids were visualized by exposure to iodine vapor, and bands corresponding to TAG, free cholesterol (FC), cholesteryl ester (CE), and phospholipid (PL) were scraped and counted using a scintillation counter. After lipid extraction, cell residue was dissolved with 0.1 N of NaOH, and protein concentrations were measured using a Pierce™ BCA Protein Assay Kit for protein normalization of data.

### Western blot

Cellular protein was harvested in Pierce™ IP lysis buffer (ThermoFisher Scientific) from Day 0 of *Abhd4* KO and WT control cells. The protein concentration of each sample was determined through the Pierce™ BCA Protein Assay Kit. A total of 20 μg cellular protein was separated on a 4%–20% polyacrylamide gel (Bio-Rad) and then transferred to a nitrocellulose membrane (Bio-Rad). The membrane was blocked using 5% non-fat milk in tris-buffered saline plus 0.1% Tween (TBST) for 2 h. Membranes were incubated overnight at 4°C in primary antibodies including ABHD4 (Sigma-Aldrich, SAB1307058) and glyceraldehyde-3-phosphate dehydrogenase (GAPDH, Santa Cruz Biotechnology, sc-477724). After incubation in primary antibodies, membranes were washed for 15 min using TBST and incubated for 1.5 h in secondary antibodies. After washing again for 15 min, the membranes were treated with the SuperSignalTM West Pico PLUS Chemiluminescent Substrate to produce a chemiluminescent signal for imaging and visualizing proteins of interest using ChemiDoc Imaging Systems (Bio-Rad).

### Cell lipolytic isoproterenol treatment

Following the procedure adapted from a previous publication (23), Day 9 differentiated 3T3-L1 *Abhd4* KO and WT control adipocytes were washed with warm DPBS twice and then cultured in Krebs-Ringer buffer, bicarbonate-buffered (Alfa Aesar), supplemented with 4% fatty acid–free BSA. Cells were then treated without (i.e., water) or with 10 μM isoproterenol (Sigma-Aldrich) to induce lipolysis for 3 h. Culture media were then collected for free fatty acid quantification using an enzymatic colorimetric assay in accordance with the manufactory procedures (Zenbio). Cells were scraped and collected for OEA quantification using mass spectrometry (See the method below). After lipid extraction, cell residue was dissolved with 0.1 N of NaOH and protein concentrations were measured using a Pierce™ BCA Protein Assay Kit for protein normalization of data.

### Mice

Mice were housed in standard cages under a 12-h light cycle and 12-h dark cycle (dark from 6:00 PM to 6:00 AM) at standard ambient temperature and humidity conditions and were provided with ad libitum water and a standard chow diet (Purina-LabDiet, Prolab RMH 3000). All experiments were performed using a protocol approved by the Institutional Animal Care and Use Committee at Wake Forest School of Medicine in facilities approved by the American Association for Accreditation of Laboratory Animal Care.

In Fig. 1B–D, 8-week-old male C57BL/6J mice (Jackson Lab #000664) were fed chow or a Western diet (Envigo #TD 88137, 42% from fat, 0.2% total cholesterol) for 16 weeks. Mice were fasted overnight before being euthanized, and tissues were collected and stored at −80°C until used. In Fig. 1E, F, 6 weeks old male C57BL/6J mice were fed chow or a high-fat diet (Research Diets Inc #D12492, 60% from fat) for 12 weeks. Epidydimal visceral white fat pads were harvested from overnight fasted mice and used for adipose tissue digestion (24). Briefly, tissue was enzymatically digested in a digestion buffer (0.5 g of fat in 10 ml) containing 0.8 mg/ml of collagenase II (Worthington Biochemicals), 3% of fatty acid free-BSA (Sigma-Aldrich), 1.2 mM of calcium chloride (Sigma-Aldrich), 1 mM of magnesium chloride (Sigma-Aldrich), and 0.8 mM of zinc Chloride (Sigma-Aldrich) in Hanks Buffered Salt Solution (Life Technologies) for 60 min in a shaking water bath at 37°C with 200 rpm agitation. The fat digest was then filtered through a 200-um filter (Fisher Scientific). The adipocyte fraction and stromal vascular (SV) fraction were collected by centrifugation at 800 *g* for 10 min. Red blood cells in the SV fraction were lysed using ACK lysis buffer. The adipocyte and SV fractions were treated with QIAzol Lysis Reagent (Qiagen) and stored at −80°C until used for gene expression. In Fig. 7, 16-week-old chow-fed male and female C57BL/6J mice were fasted for 24 h or fasted for 24 h and then refed chow for 12 h. Male and female gonadal visceral white and interscapular brown adipose tissues were collected for gene expression and OEA quantification. Mouse serum samples were also collected for quantifying free fatty acid concentrations (Zenbio).

In Figs. 5 and 8, heterozygous *Abhd4*^+/−^ mice (#46224-JAX) were purchased from the Jackson Lab and crossed to generate whole-body *Abhd4* KO (*Abhd4*^−/−^) and their WT (*Abhd4*^+/+^) littermate control mice. Mouse genotype was determined using a genotyping protocol (Protocol 34917) provided by the Jackson Lab. About 6–7 weeks old male and female *Abhd4*^−/−^ and *Abhd4*^+/+^ mice were fed a high-fat diet (i.e., Research Diets, 45% kcal from fat) to induce obesity. Mouse body weights were recorded weekly. Mice were used for systemic metabolic phenotyping including 1) serial measurements of mouse body composition (ie, whole body fat, lean, free water, and total water masses in live animals) using EchoMRI™, 2) glucose and insulin tolerance tests (22): Mice were fasted for 16 h for an intraperitoneal glucose (Sigma-Aldrich) tolerance test (1 g/kg body weight) and fasted for 4 h for intraperitoneal insulin (Eli Lilly) tolerance test (0.75 U/kg body weight), and 3) indirect calorimetry (21): Metabolic cages (TSE PhenoMaster system) were used in awake mice to simultaneously measure oxygen consumption, carbon dioxide production, respiratory exchange ratio, energy expenditure, food/water intake, and activity during a 12-h light/12-h dark cycle for 5 consecutive days. Mice were fasted overnight before being euthanized, and their serum samples were collected to measure serum free fatty acid and insulin levels. Mouse tissues were weighted, snap-frozen in liquid nitrogen, and stored at −80°C until used for gene expression and NAE quantification.

A portion of epidydimal visceral white fat pads was used for adipose tissue digestion as described above (24). After discarding the adipocyte fraction, the SV cell fraction was resuspended in the medium for proliferation and differentiation into adipocytes at a higher concentration of dexamethasone at 1 μM (Day 0–3) and insulin at 2 μg/ml (Day 0–9).

An floxed *Abhd4* (*Abhd4*^flox/flox^) mouse model was generated by flanking exons 3–4 of *Abhd4* gene locus and an inverted reporter cassette of 3_SA_IRES_eGFP (3_Splice Acceptor_Internal Ribosome Entry Site_enhanced Green Fluorescent Protein), with loxP and loxP_2272 sites, via gene targeting in C57BL/6 mouse embryonic stem cells (supplemental Fig. S1B) and provided by Ozgene (mice are available upon request). Cre recombinase-mediated deletion of the floxed region should excise exons 3–4 and invert the GFP knockin cassette (3_SA_IRES_eGFP) into the correct orientation for expression (supplemental Fig. S1C). Exons 3 and 4 encode part of the alpha/beta hydrolase fold-1 and loss of this protein domain should eliminate hydrolase activity. In addition, along with the deletion of exon 3 and 4, transcription and translation should be terminated with the inclusion of GFP-PolyA cassette, resulting in the expression of a truncated protein. Thus, cells lacking *Abhd4/*ABHD4 expression can be identified by real-time PCR/Western blot and GFP detection using microscopy.

*Abhd4*^flox/flox^ mice were crossed with hemizygous adiponectin-Cre (*Adipoq*-Cre^+/−^) mice (Jackson Lab #028020) (25) for the creation of adipocyte-specific *Abhd4* KO (*Abhd4*^flox/flox^*Adipoq*-Cre^+/−^ as *Abhd4*^adipose−/−^) and their littermate control (*Abhd4*^flox/flox^*Adipoq*-Cre^−/−^ as *Abhd4*^adipose+/+^). The mouse genotype was determined using genotyping protocols provided by Ozgene (Table 1 for *Abhd4*^flox/flox^) and the Jackson Lab (Protocol #20627 for *Adipoq*-Cre^+/−^) on the 7500 Real-Time PCR Systems.

**Table 1:** Primers for genotyping a floxed *Abhd4* mouse

**1. 2156_Lo5WT**	Amplifies the wt Allele Across the Site at which the 5′ loxP is to Be Inserted
PrimeTime Primer 1 (5′-3′)	GTCTGCCATGGTGTGTTATG
PrimeTime Primer 2 (5′-3′)	GAGTGAATTTTGTGTGACAACC
PrimeTime Probe (5′-3′)	CAATGGTGGCTACTGCCCTGAGTC
Dye-Quencher Mod	FAM/ZEN/IBFQ
Genotypes	wt/wt - 2 copies; wt/flox - 1 copy; flox/flox - 0 copy

**2. 2156_LoWT3**	Amplifies the wt allele across the site at which the 3′ loxP is inserted
PrimeTime Primer 1 (5′-3′)	AGAATTCCTGGGTAAAGCATC
PrimeTime Primer 2 (5′-3′)	GAGGGCTTCCTAAAATGATCT
PrimeTime Probe (5′-3′)	CTTCCTGAGAGCACAGACATTTTGCC
Dye-Quencher Mod	FAM/ZEN/IBFQ
Genotypes	wt/wt - 2 copies; wt/flox - 1 copy; flox/flox - 0 copy

**3. 2156_GoConK**	Amplifies the floxed allele
PrimeTime Primer 1 (5′-3′)	TGGGCTCTATGGTGCAT
PrimeTime Primer 2 (5′-3′)	TCTGATGTGCCACTTCTC
PrimeTime Probe (5′-3′)	ATCTACGTGCGTCACATGCAGTAC
Dye-Quencher Mod	FAM/ZEN/IBFQ
Genotypes	wt/wt - 0 copy; wt/flox - 1 copy; flox/flox - 2 copies

**4. TERT_qPCR**	Amplifies Tert on Chr13. MGI: 1202709 and is used for assay normalization
PrimeTime Primer 1 (5′-3′)	GAGACAATGGGTGGCAGTAA
PrimeTime Primer 2 (5′-3′)	GCTTGGAGTCAGAGACCATAAG
PrimeTime Probe (5′-3′)	ATGCAGTCCGTGGTTGGATGAGTT
Dye-Quencher Mod	HEX/ZEN/IBFQ or JOE/ZEN/IBFQ

In Figs. 6, 8, and S2, 8 weeks old male and female *Abhd4*^adipose−/−^ and *Abhd4*^adipose+/+^ mice were maintained on chow or fed a high-fat diet (i.e., Research Diets, 45% kcal from fat) to induce obesity. Mouse body weights were recorded weekly. Mice were used for systemic metabolic phenotyping and then euthanized to collect serum samples and tissues as described above. In addition, a portion of epidydimal visceral white fat pads was used for adipose tissue digestion as described above (24).

### Mouse lipolytic CL-316,243 injection

Mouse blood samples (∼100 μl) were collected in the fed state, at ∼10:00 AM, and then mice were injected intraperitoneally a bolus of CL-316,243 (Sigma-Aldrich) at 1 mg/kg body weight (26). Two hours following CL-316,243 injection, mouse blood samples (∼100 μl) were collected for quantifying free fatty acid concentrations (Zenbio).

### Mass spectrometry

Cells were collected and tissues were weighed out into a homogenization tube with 2.8 mm ceramic beads (Fisher Scientific) before the addition of 1 ml of methanol and 10 μl of internal standard solution (200 pg/μl OEA-d4 and PEA-d4). The samples were then homogenized using a Bead Ruptor 24 (OMNI International) for 3 cycles of 20 s. These homogenized samples were then centrifuged for 10 min at 16,000xg. Supernatants were transferred into glass vials and diluted with 4 ml of 0.1% formic acid, then mixed thoroughly. After lipid extraction, cell/tissue residue was dissolved with 0.1 N of NaOH, and protein concentrations were measured using a Pierce™ BCA Protein Assay Kit for protein normalization of data.

Reverse-phase cartridges (Waters Sep-Pak Vac 1 cc (100 mg) tC18) were activated with 1 ml of methanol and then equilibrated with 3 ml of 0.1% formic acid. Samples were then loaded onto the columns. Columns were rinsed twice, first with 1 ml of 0.1% formic acid, then with 1 ml of 15% methanol. Samples were finally eluted with 2 steps of methanol in 500 μl aliquots.

OEA, AEA, and PEA were quantified using either a Thermo Q Exactive HF hybrid quadrupole-Orbitrap mass spectrometer or a Shimadzu UHPLC-MS/MS. The Q Exactive HF was equipped with a heated electrospray ion source and a Vanquish UHPLC System. The Shimadzu instrument was equipped with Nexera UHPLC system and an 8050 triple-quadrupole mass spectrometer utilizing a DUIS source.

Chromatographic methods were identical for both instruments, and the compounds were separated on either instrument with a Kinetex C8 (Phenomenex, 150 × 3 mm, 2.6 μm) with a flow rate of 0.4 ml/minute and mobile phases consisting of 0.1% formic acid for mobile phase A and acetonitrile for mobile phase B. The mobile phase gradient began at 30% B and was then increased to 95% B at 9 min. This percentage was held for 2 min before being decreased to 30% B at 11.1 min and then held at that final percentage until 15 min.

Ionization occurred at the DUIS source set to the following parameters: nebulizing gas flow of 2 L/min, heating gas flow of 10 L/min, interface temperature of 300°C, DL temperature of 250°C, heat block temperature of 400°C, and a drying gas flow of 10 L/min. Each analyte and its deuterated internal standard used unique MRM transitions in positive ESI mode: OEA 330.20>66.10 and OEA-d4 326.20>62.10; PEA 300.10>62.10, 44.15, 287.25 and PEA-d4 304.10, 44.15, 287.25; AEA 348.2>287.264, 62.046, 44.069.

### Statistics

Data were presented as mean ± standard error of the mean throughout the figures. All data points reflected biological replicates. Binary comparisons were performed using a two-tailed Student's unpaired *t* test. Datasets comparing the effect of a single independent variable on more than two groups were assessed by one-way ANOVA followed by Dunnett's multiple comparisons. Datasets containing groups defined by two independent variables were assessed by two-way ANOVA with Sidak’s multiple comparisons. Prism 7 software (GraphPad) is used to perform statistical analyses (Statistical significance *P* < 0.05) and generate graphical representations of data. CalR, a web-based tool, was used to perform statistical analyses (Statistical significance *P* < 0.05) and generate graphical representations of indirect calorimetry (TSE PhenoMaster system) data (27).

## Results

### The mRNA expression of *Abhd4* in adipose tissue is positively associated with obesity

We first examined gene expression of *Nape-pld*, *Abhd4*, *Gde1*, *Gde7/Gdpd3*, and *Gde4/Gdpd1* in adipose tissues of obese mice. We found that Western diet-induced obese mice versus chow-fed lean mice (Fig. 1B) had increased *Abhd4* mRNA levels in white (Fig. 1C) and brown (Fig. 1D) adipose tissue. There was no difference in adipose *Nape-pld*, *Gde1*, *Gde7/Gdpd3, Gde4/Gdpd1* mRNA levels between lean and obese mice (Data not shown).

Cellular components of adipose tissue include mature adipocyte fraction and stromal vascular (SV) cell fraction which consists of immune cells, fibroblasts, vascular cells, multipotent mesenchymal stem cells, and adipocyte precursor cells (28). Thus, we sought to determine which fraction was responsible for the observed increase in *Abhd4* mRNA levels in the adipose tissue of diet-induced obese mice compared to lean mice. We found that SV and mature adipocyte fractions showed similar levels of *Abhd4* mRNA, which increased in both fractions (Fig. 1E) isolated from epididymal white adipose tissue of a high fat diet-induced obese mice (body weight at 48.3 ± 1.7 g) compared to chow-fed lean mice (body weight at 29.3 ± 0.6 g, *P* < 0.0001). Adipocyte fraction was identified by the abundance of peroxisome proliferator-activated receptor gamma (*Pparγ*) (Fig. 1F), which is highly expressed in adipose tissues and markedly induced during adipogenesis (29).

Furthermore, we examined whether *Abhd4* gene expression was altered during adipogenesis using a wildtype (WT) 3T3-L1 mouse pre-adipocyte cell line. During adipogenesis, fibroblast-like pre-adipocytes differentiate into lipid-laden and insulin-responsive adipocytes. Adipogenesis is a complex and multi-step process involving the activation of a cascade of transcription factors such as CCAAT/enhancer binding proteins (C/EBPs), PPARs, and sterol regulatory element binding proteins (SREBPs) that induce gene expression including acetyl-CoA carboxylase 1 (ACC1) and fatty acid synthase (FASN) for de novo fatty acid synthesis, fatty acid-binding protein 4 (FABP4) for fatty acid chaperones, CD36 for fatty acid uptake, as well as glycerol phosphate acyltransferase (GPAT), acylglycerolphosphate acyltransferase (AGPAT), and diacylglycerol acyltransferase (DGAT) for fatty acid esterification to form TAG, all of which lead to adipocyte development (30, 31). We observed *Abhd4* mRNA expression was high in WT 3T3-L1 pre-adipocytes (Day 0) and throughout adipocyte differentiation (Day 3–9) with an average Ct value of 27.0–28.8 and their endogenous control gene 18s average Ct was 16.0–16.7 (Fig. 1G). Please note that, in comparison with the master regulator of adipogenesis, *Pparγ*, we observed that Day 9 differentiated WT 3T3-L1 adipocytes had an average Ct value of 26.9 and their endogenous control gene 18s had an average Ct value of 15.7.

**Fig. 2:**
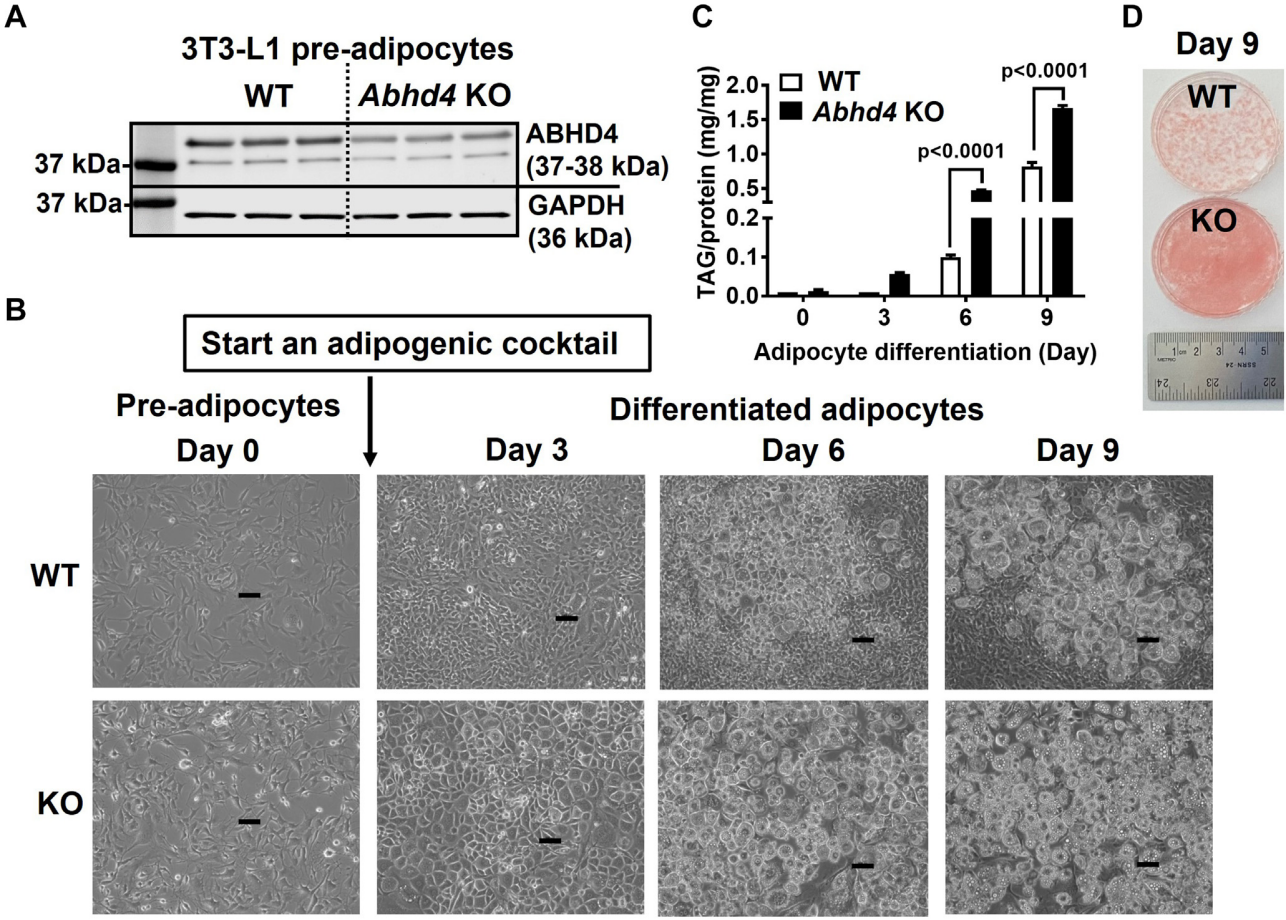
The effect of *Abhd4* deletion on adipocyte differentiation. A: Cellular proteins of wildtype (WT) control and *Abhd4* knockout (KO) 3T3-L1 pre-adipocytes (n = 3/genotype) were harvested and subjected to Western blot using anti-ABHD4 and anti-GAPDH antibodies. B: WT and *Abhd4* KO 3T3-L1 cells (n = 3/genotype) were proliferated for 2 days (Day 0) and then differentiated into adipocytes for 9 days. Microscopic images at 10X magnification were captured using a phase contrast microscope with a scale of 100 μm. C: Day 0, 3, 6, and 9 cells were lipid-extracted to measure triacylglycerol (TAG) mass by a colorimetric assay. Results are analyzed using a two-way ANOVA with Sidak multiple comparisons. D: The TAG in Day 9 cells was stained with Oil Red O and photographed.

Overall, because 1) there is a positive correlation between adipose tissue *Abhd4* gene expression and adiposity/obesity in mice (Fig. 1B–D), and 2) *Abhd4* mRNA was abundant in both SV cell and adipocyte fractions of adipose tissue (Fig. 1E, F) as well as in both 3T3-L1 pre-adipocytes and newly differentiated mature adipocytes (Fig. 1G), we chose a loss-of-function approach instead of a gain-of-function approach to better understand the functional role of *Abhd4* in adipocyte differentiation and lipid biology.

### Deletion of *Abhd4* promotes adipogenesis in vitro

We have generated an *Abhd4* KO 3T3-L1 pre-adipocyte and observed that a small amount of truncated ABHD4 protein was detected by Western blot in 3T3-L1 *Abhd4* KO compared to WT control pre-adipocytes (Fig. 2A). We found that during adipogenic stimuli, *Abhd4* KO cells acquired adipocyte morphology and accumulate TAG faster than WT control cells (Fig. 2B); TAG content was ten-fold higher in *Abhd4* KO than in WT cells at Day 3, five-fold higher at Day 6 (*P* < 0.0001), and two-fold higher at Day 9 (*P* < 0.0001) of adipocyte differentiation (Fig. 2C). The TAG in Day 9 adipocytes was stained with Oil Red O (Fig. 2D). The WT cells showed typical 3T3-L1 cell culture morphology with a cluster of adipocytes showing red color surrounded by non-differentiated, non-stained fibroblast-like cells (Fig. 2D). All of *Abhd4* KO adipocytes had TAG lipid droplets on Day 9 (Fig. 2D). These data suggest that *Abhd4* deletion in 3T3-L1 cells increases adipogenesis and lipid accumulation.

**Fig. 3:**
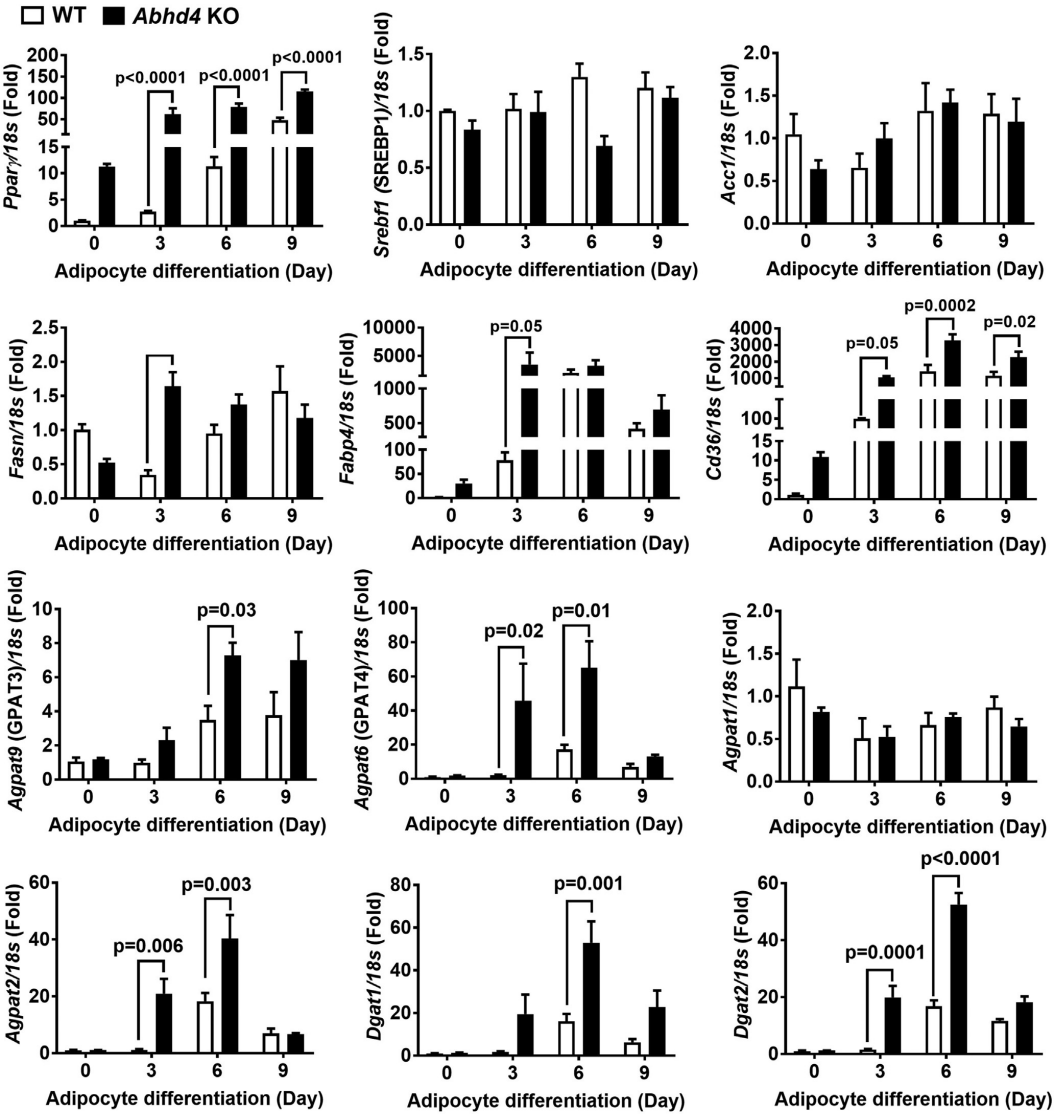
The effect of *Abhd4* deletion on adipogenic and lipogenic gene expression. Cellular RNA was extracted from wildtype (WT) control and *Abhd4* knockout (KO) 3T3-L1 cells (n = 3/genotype) and reverse-transcribed into cDNA for real-time PCR quantification of *Pparγ*, *Srebf1*, *Acc1*, *Fasn*, *Fabp4*, *Cd36, Agpat9, Agpat6, Agpat1, Agpat2, Dgat1, and Dgat2* normalized to *18s* (endogenous control). Results are presented as the fold change compared to WT at Day 0 and analyzed using a two-way ANOVA with Sidak multiple comparisons.

We then examined several key genes that play a role in adipogenesis and lipid metabolism. The *Abhd4* KO versus WT control adipocytes have increased mRNA levels of transcription factor *Pparγ*, but not *C/ebp* (Data not shown) and *Srebf* (i.e., SREBP1) family, during adipocyte differentiation (Fig. 3). In addition, except *Acc1* and *Agpat1*, the mRNA levels of *Fasn*, *Fabp4*, *Cd36, Agpat9* (i.e., GPAT3), *Agpat6* (i.e., GPAT4), *Agpat2*, *Dgat1*, and *Dgat2* were increased in *Abhd4* KO compared to WT control adipocytes during Day 3–9 of differentiation (Fig. 3).

### Deletion of *Abhd4* promotes lipogenesis in vitro

**Fig. 4:**
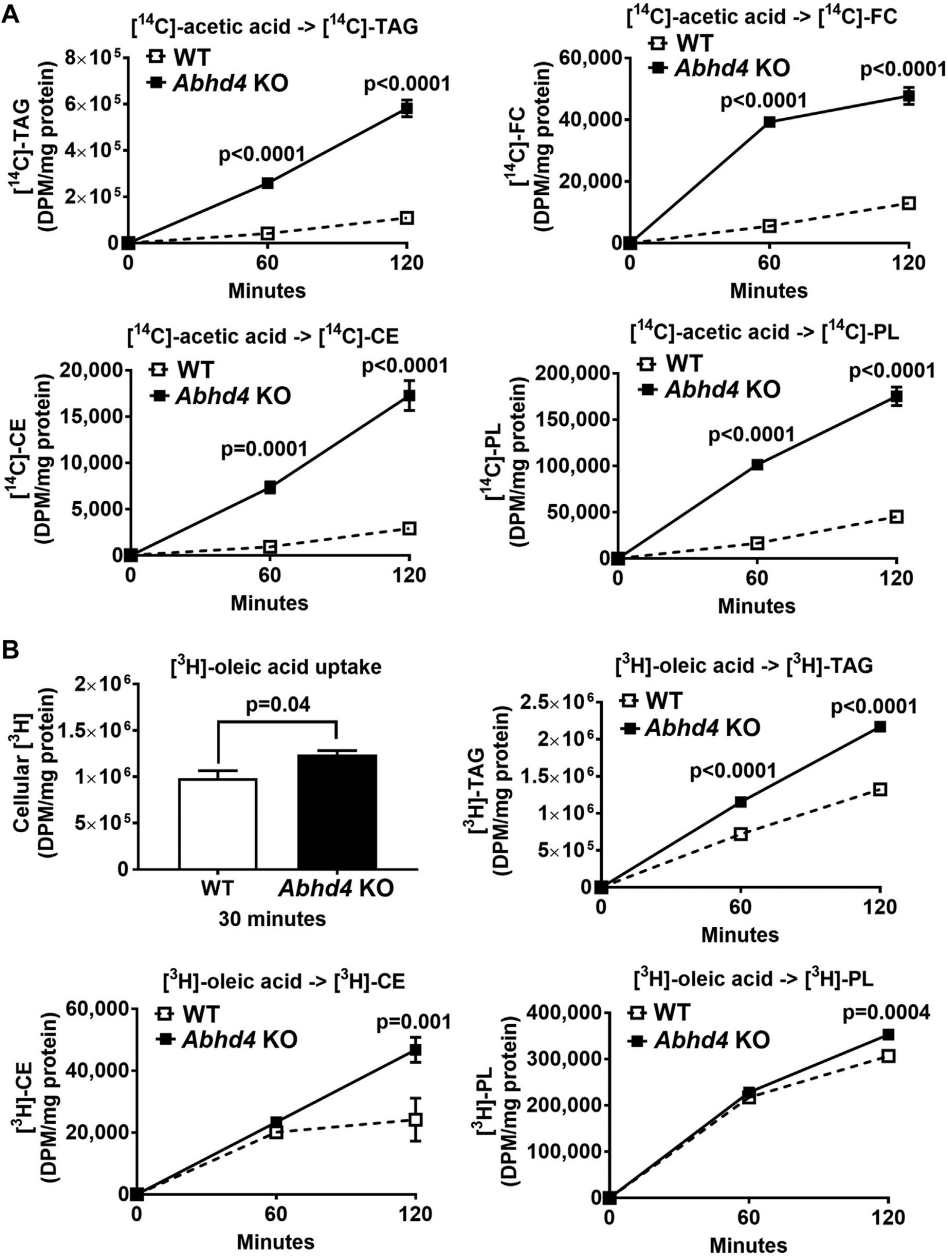
The effect of *Abhd4* deletion on lipogenesis. Day 7 differentiated wildtype (WT) control and *Abhd4* knockout (KO) 3T3-L1 adipocytes (n = 3/genotype) were treated with (A) 0.5 μCi/ml of [1,2–^14^C]-acetic acid or (B) 5 μCi/ml of [9,10-^3^H(N)]-oleic acid at the indicated times. Cells were lipid-extracted, and triacylglycerol (TAG), free cholesterol (FC), cholesteryl ester (CE), and phospholipid (PL) were separated using thin layer chromatography. [^14^C]-TAG, [^14^C]-FC, [^14^C]-CE, [^14^C]-PL, cellular [^3^H], [^3^H]-TAG, [^3^H]-CE, and [^3^H]-PL were quantified by liquid scintillation counting. Results are analyzed using a two-way ANOVA with Sidak multiple comparisons (i.e., [^14^C]-TAG, [^14^C]-FC, [^14^C]-CE, [^14^C]-PL, [^3^H]-TAG, [^3^H]-CE, and [^3^H]-PL) and a two-tailed Student's unpaired *t* test (i.e., cellular [^3^H]).

Next, we performed functional characterization. Day 7 differentiated WT control and ABHD4-deficient adipocytes were treated with [^14^C]-acetic acid to determine de novo lipogenesis or [^3^H]-oleic acid to measure fatty acid uptake and esterification. The rate of TAG, free cholesterol (FC), cholesteryl ester (CE), and phospholipid (PL) synthesis from [^14^C]-acetic acid was significantly increased (*P* < 0.0001) in ABHD4-deficient adipocytes versus WT control adipocytes (Fig. 4A). ABHD4-deficient adipocytes also had significantly increased cellular uptake of [^3^H]-oleic acid (*P* = 0.04) and incorporation of [^3^H]-oleic acid into TAG (*P* < 0.0001), CE (*P* = 0.001), and PL (*P* = 0.0004) compared to WT control adipocytes (Fig. 4B). These data demonstrate that ABHD4 deficiency increases lipogenesis, supporting the cell phenotype and gene expression results.

### High fat–fed male and female whole-body *Abhd4* KO mice have a similar metabolic phenotype as their control littermates

**Fig. 5:**
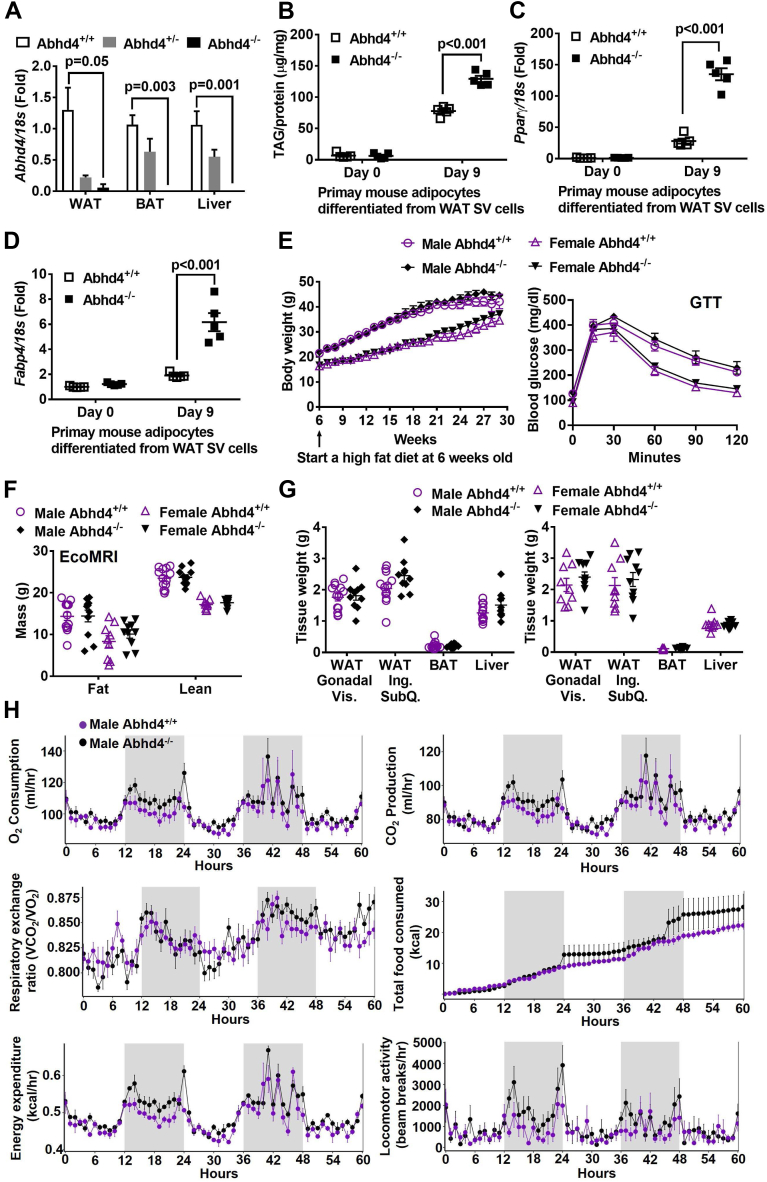
Metabolic phenotype of whole body *Abhd4* knockout (*Abhd*4^−/−^) versus their control littermate (*Abhd4*^+/+^) mice. A: Epididymal visceral white adipose tissue (WAT), interscapular brown adipose tissue (BAT), and liver were collected from 8 weeks old male *Abhd4*^+/+^, *Abhd4*^+/−^, and *Abhd4*^−/−^ mice (n = 3–7) for RNA extraction, cDNA synthesis, and real-time PCR quantification of *Abhd4* normalized to *18s* (endogenous control). Results are presented as the fold change compared to *Abhd4*^+/+^ and analyzed using a one-way ANOVA with Dunnett's multiple comparisons. B: Stromal vascular (SV) cells were isolated from epididymal visceral WAT of chow-fed male mice (n = 5/genotype), differentiated into adipocytes for 9 days, and then lipid-extracted to measure triacylglycerol (TAG) mass by a colorimetric assay. Cellular RNA was extracted from another set of adipocytes and reverse-transcribed into cDNA for real-time PCR quantification of (C) *Pparγ* and (D) *Fabp4* normalized to *18s* (endogenous control). Results are presented as the fold change compared to *Abhd4*^+/+^ cells at Day 0. E: Six weeks old male (n = 10–13/genotype) and female (n = 9–10/genotype) mice were fed a high fat diet and their body weight was measured weekly. After a 16-weeks high fat diet feeding, at 22 weeks of age, mice were fasted for 16 h, and glucose tolerance test (GTT) was conducted. Results are analyzed using a two-way ANOVA with Sidak multiple comparisons. B–E: Data are analyzed using a two-way ANOVA with Sidak multiple comparisons. F: Body composition of mice fed a high fat diet for 15 weeks, at 21 weeks of age, were quantified by EcoMRI. G: After mice were on a high fat diet for 24 weeks, at 30 weeks of age, their gonadal visceral (Vis.) WAT, inguinal (Ing.) subcutaneous (SubQ.) WAT, BAT, and liver were then collected and weighted. F–G: Data are analyzed using a two-tailed Student's unpaired *t* test. H: After a 17-weeks of a high fat diet feeding, at 23 weeks of age, male mice (n = 3/genotype) were used for indirect calorimetry (TSE PhenoMaster System). The graphs were created, and statistical analysis (one-way ANOVA GLM) was performed using a software package, CalR.

We then investigated whether deletion of *Abhd4* in fetal development promotes adipogenesis and lipogenesis in vivo by using whole-body *Abhd4* KO (*Abhd4*^−/−^) mice and their control littermates (*Abhd4*^+/+^). Mouse genotype was verified by standard PCR and quantitative real-time PCR (Fig. 5A). In a similar fashion to data obtained from *Abhd4* KO 3T3-L1 (Figs. 2 and 3), at Day 9 of adipocyte differentiation, SV cells isolated from the epidydimal visceral white adipose tissue of chow-fed *Abhd4*^−/−^ mice accumulated more TAG (Fig. 5B) and showed higher *Pparγ* (Fig. 5C) and *Fabp4* (Fig. 5D) mRNA levels than *Abhd4*^+/+^ mice. However, we observed no difference in body weight and glucose tolerance test (GTT) between male or female *Abhd4*^−/−^ and *Abhd4*^+/+^ mice on chow (Data not shown) or a high-fat diet (Fig. 5E). There was also no difference in mouse fat and lean mass quantified by EcoMRI between male or female *Abhd4*^−/−^ and *Abhd4*^+/+^ mice (Fig. 5F). Furthermore, the individual fat pad mass was unaffected by ABHD4 deficiency (Fig. 5G). Among indirect calorimetry measurements including oxygen consumption, carbon dioxide production, respiratory exchange ratio, food intake, energy expenditure, locomotor activity, and ambulatory activity (Data not shown), interestingly, male *Abhd4*^−/−^ mice consumed more food (kcal, *P* = 0.009) but also had increased energy expenditure (kcal/hr, *P* = 0.04) compared to their littermate control male *Abhd4*^+/+^ mice (Fig. 5H), thus the body weight/composition did not differ between the mouse genotypes (Fig. 5E–G). Contrary to male mouse indirect calorimetry results, female mice exhibited similar patterns of food intake and energy expenditure (data not shown).

### Both male and female adipocyte-specific *Abhd4* KO mice have a similar metabolic phenotype as their littermate controls on chow or a high-fat diet

**Fig. 6:**
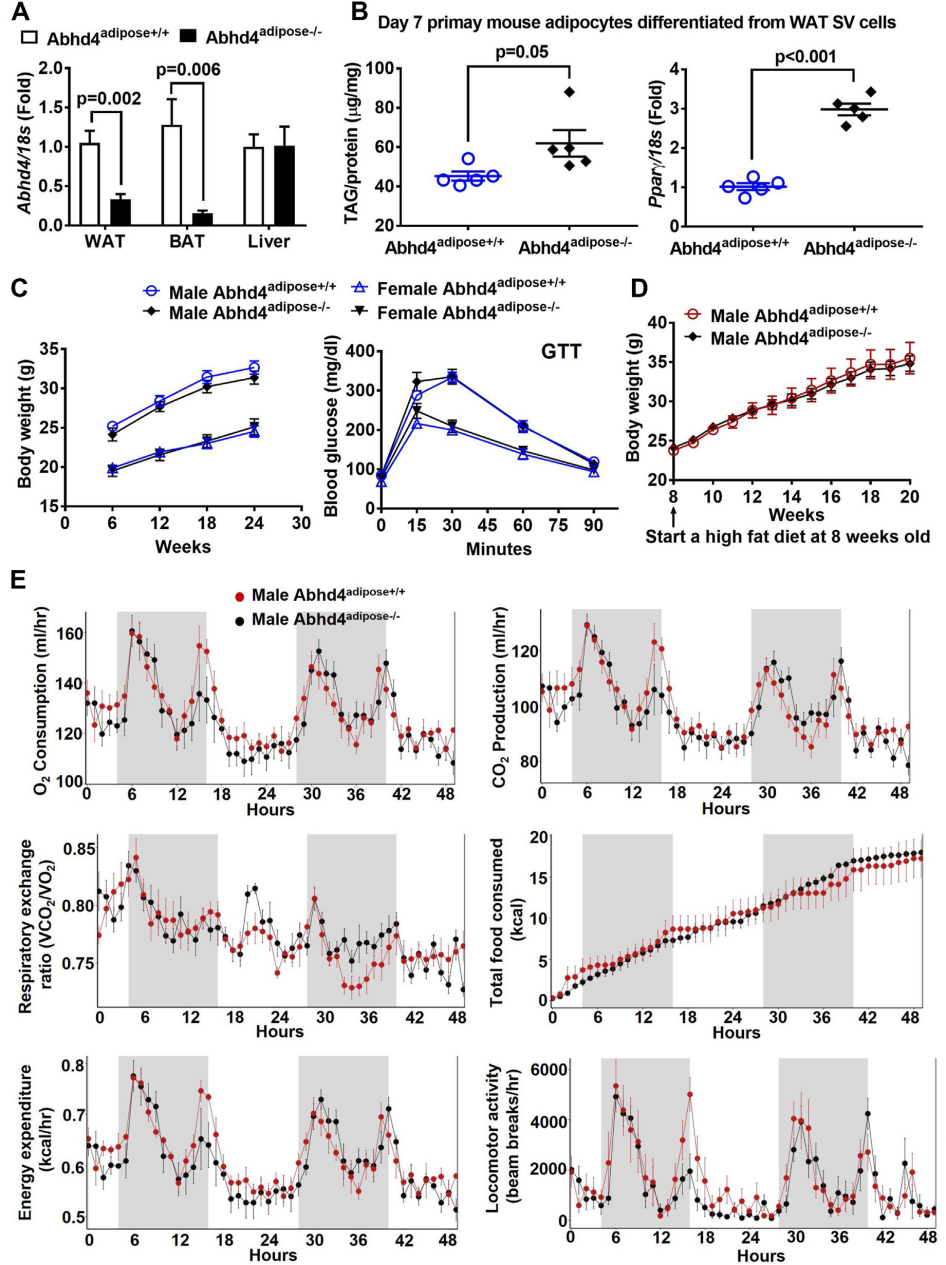
Metabolic phenotype of adipocyte specific *Abhd4* knockout (*Abhd4*^adipose−/−^) versus their control littermate (*Abhd4*^adipose+/+^) mice. A: Gonadal visceral white adipose tissue (WAT), brown adipose tissue (BAT), and liver were harvested from 8 weeks old male and female *Abhd4*^adipose+/+^ and *Abhd4*^adipose−/−^ mice (n = 3/genotype/sex) for RNA extraction, cDNA synthesis, and real-time PCR quantification of *Abhd4* normalized to *18s* (endogenous control). Results are presented as the fold change compared to *Abhd4*^adipose+/+^. B: Stromal vascular (SV) cells were isolated from epididymal visceral WAT of chow-fed male mice (n = 5/genotype), differentiated into adipocytes for 7 days, and then lipid-extracted to measure triacylglycerol (TAG) mass by a colorimetric assay. Cellular RNA was extracted from another set of adipocytes and reverse-transcribed into cDNA for real-time PCR quantification of *Pparγ* normalized to *18s* (endogenous control). Results are presented as the fold change compared to *Abhd4*^adipose+/+^. A, B: Data are analyzed using a two-tailed Student's unpaired *t* test. C: Chow-fed mice (n = 7–9/sex/genotype) were weighted weekly, and data were presented every 6 weeks. Mice were fasted for 16 h, and glucose tolerance test (GTT) was conducted. D: Eight weeks old male mice (n = 7–8/genotype) were fed a high fat diet and their body weights were measured weekly. C, D: Results are analyzed using a two-way ANOVA with Sidak multiple comparisons. E: After a 12-weeks of a high fat diet feeding, at 20 weeks of age, male mice (n = 3/genotype) were used for indirect calorimetry (TSE PhenoMaster System). The graphs were created, and statistical analysis (one-way ANOVA GLM) was performed using a software package, CalR.

We wanted to further investigate whether deletion of *Abhd4* in adipocytes disrupts lipid homeostasis and systemic metabolism by crossing *Abhd4*^flox/flox^ mice with hemizygous adiponectin-Cre (*Adipoq*-Cre^+/−^) mice to produce adipocyte-specific *Abhd4* KO (*Abhd4*^flox/flox^*Adipoq*-Cre^+/−^ as *Abhd4*^adipose−/−^) mice and their control littermates (*Abhd4*^flox/flox^*Adipoq*-Cre^−/−^ as *Abhd4*^adipose+/+^). Adiponectin expression increases 4 to 6 days after adipocyte differentiation and is considered a marker of mature adipocytes (32, 33). Mouse genotype was verified by standard PCR and quantitative real-time PCR (Fig. 6A). Approximately 20%–30% of *Abhd4* mRNA was detected in adipose tissues of *Abhd4*^adipose−/−^ mice (Fig. 6A). The SV cells of the adipose tissue are likely responsible for this (∼20%–30% of *Abhd4* mRNA was detected in adipose tissues of *Abhd4*^adipose−/−^ mice), since we found *Abhd4* mRNA abundance in the SV cell fraction of WT mice (Fig. 1E). Similar to the data obtained from *Abhd4* KO 3T3-L1 (Figs. 2 and 3) and *Abhd4*^−/−^ (Fig. 5B–D), at Day 7 of adipocyte differentiation, SV cells isolated from epidydimal white visceral adipose tissue of *Abhd4*^adipose−/−^ mice had an increase in TAG and *Pparγ* compared to *Abhd4*^adipose+/+^ mice on chow (Fig. 6B). However, there was no difference in body weights and metabolic outcomes such as GTT between male or female *Abhd4*^adipose−/−^ and *Abhd4*^adipose+/+^ mice on chow (Fig. 6C). Then, mice were given a high-fat diet. There was no difference in body weight (Fig. 6D), GTT (Data not shown), and insulin tolerance test (Data not shown) as well as indirect calorimetry measurements (Fig. 6E) between *Abhd4*^adipose−/−^ and *Abhd4*^adipose+/+^ mice on a high-fat diet. Please note that despite mice having the C57BL/6 background, well known for being susceptible to diet-induced obesity, we only observed a mild weight gain in our male mice (Fig. 6D) and female mice were resistant to diet-induced obesity (Data not shown). Collectively, these data suggest that deletion of *Abhd4* in fetal development (*Abhd4*^−/−^) or in adipocytes (*Abhd4*^adipose−/−^) does not affect adipose and systemic metabolism in mice (both male and female) fed chow or a high-fat diet.

### Adipose *Abhd4* expression correlates with adipose oleoylethanolamide production and lipolysis in mice during fasting

Since ABHD4 plays a role in NAE production (Fig. 1A), we wanted to investigate whether deletion of *Abhd4* in adipocytes affects NAE such as oleoylethanolamide (OEA) levels in adipose tissues of mice. The rationale to study OEA is because Piomelli and colleagues discovered that feeding induces intestinal NAPE-PLD expression and activity, which cleaves NAPE (Fig. 1A) such as *N*-oleoyl-phosphatidylethanolamine (NOPE) to produce OEA, one of NAE molecules (34, 35). PPARα is activated by OEA, resulting in gut vagal afferent stimulation and satiety (36, 37). They also found that endogenous OEA production exhibits a diurnal cycle in rat white adipose tissue (i.e., higher OEA levels during the daytime or fasting since mice are nocturnal) (38), in contrast to feeding-induced OEA production in the gut, suggesting tissue-specific stimuli may affect OEA production. Furthermore, exogenous OEA treatment of freshly isolated rat adipocytes or in vivo treatment of rats and mice with OEA stimulates lipolysis, which does not occur in adipocytes or mice lacking PPARα (39). However, enzymes that control adipose OEA turnover and OEA-mediated lipolysis in response to a diurnal cycle or fasting status have not been characterized.

**Fig. 7:**
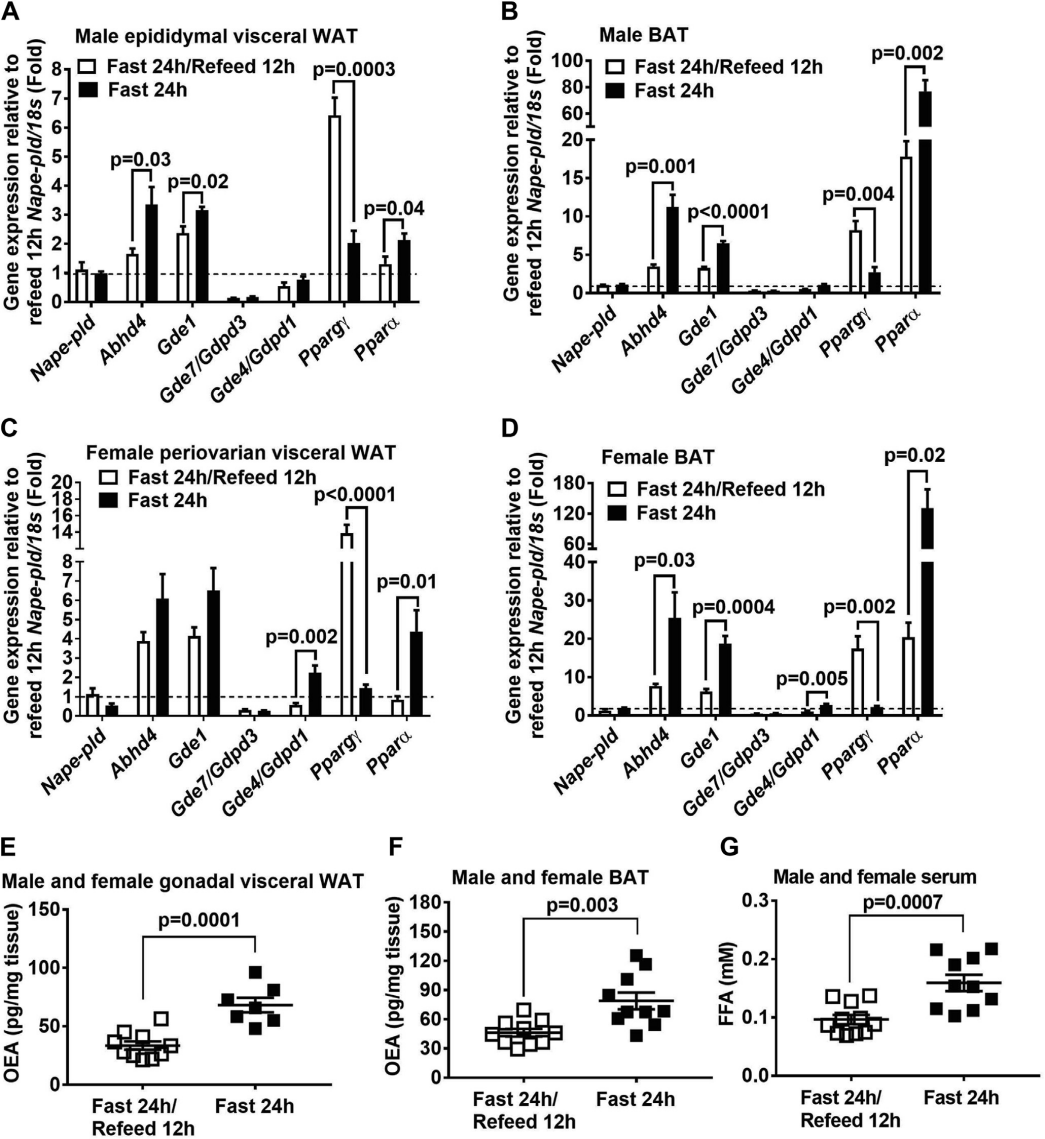
An association between adipose *Abhd4* expression, oleoylethanolamide (OEA) production, and lipolysis. Sixteen weeks old (A, B) male and (C, D) female C57BL/6J mice were fasted for 24 h (n = 5/sex) or fasted for 24 h and then refed chow for 12 h (n = 5/sex). (A, C) RNA was extracted from gonadal visceral white adipose tissue (WAT) and (B, D) interscapular brown adipose tissue (BAT) and reverse-transcribed into cDNA for real-time PCR quantification of *Nape-pld*, *Abhd4, Gde1, Gde7/Gdpd3*, *Gde4/Gdpd1*, *Pparα*, and *Pparγ* normalized to *18s* (endogenous control). Results are presented as fold change relative to *Nape-pld/18s* of 12-h refeeding. E: Gonadal visceral WAT (n = 7–10) and (F) BAT (n = 10) was used for quantifying OEA content using mass spectrometry. G: Serum free fatty acid (FFA) concentrations were measured using an enzymatic colorimetric assay. Results are analyzed using a two-tailed Student's unpaired *t* test.

We first performed a fasting and refeeding study to investigate the association between the gene expression of enzymes in the classical pathway (i.e., *Nape-pld*) versus the alternative pathway (i.e., *Abhd4, Gde1, Gde7/Gdpd3*, and *Gde4/Gdpd1*), and the production as well as function of OEA in adipose tissues. We found that *Abhd4* and *Gde1* (i.e., the alternative pathway), but not *Nape-pld* (i.e., the classical pathway), mRNA levels were increased in epididymal visceral white (Fig. 7A) and interscapular brown (Fig. 7B) adipose tissue of chow-fed male C57BL/6J mice after a 24-h fast compared to 24-h fasting/12-h refeeding. A similar trend in adipose gene expression was also observed in chow-fed female C57BL/6J mice (Fig. 7C, D). The mRNA level of *Gde7/Gdpd3* was relatively low in both adipose tissues (Fig. 7A–D). Fasting and refeeding regulate *Pparα* and *Pparγ* gene expression in adipose tissues, so these genes were used as a validation here (40, 41). We further found that fasting versus refeeding induced OEA production in both white (Fig. 7E) and brown (Fig. 7F) adipose tissues, and enhanced lipolysis, indicated by elevated circulating free fatty acid concentrations (Fig. 7G). These data demonstrate that the alternative pathway is predominant in adipose tissues (i.e., relatively high mRNA levels of *Abhd4* and *Gde1*), and there is a positive association between adipose *Abhd4* expression and OEA production and lipolysis in the fasted state.

### Deletion of *Abhd4* decreases lipolysis capacity *in vitro* but not *in vivo*

**Fig. 8:**
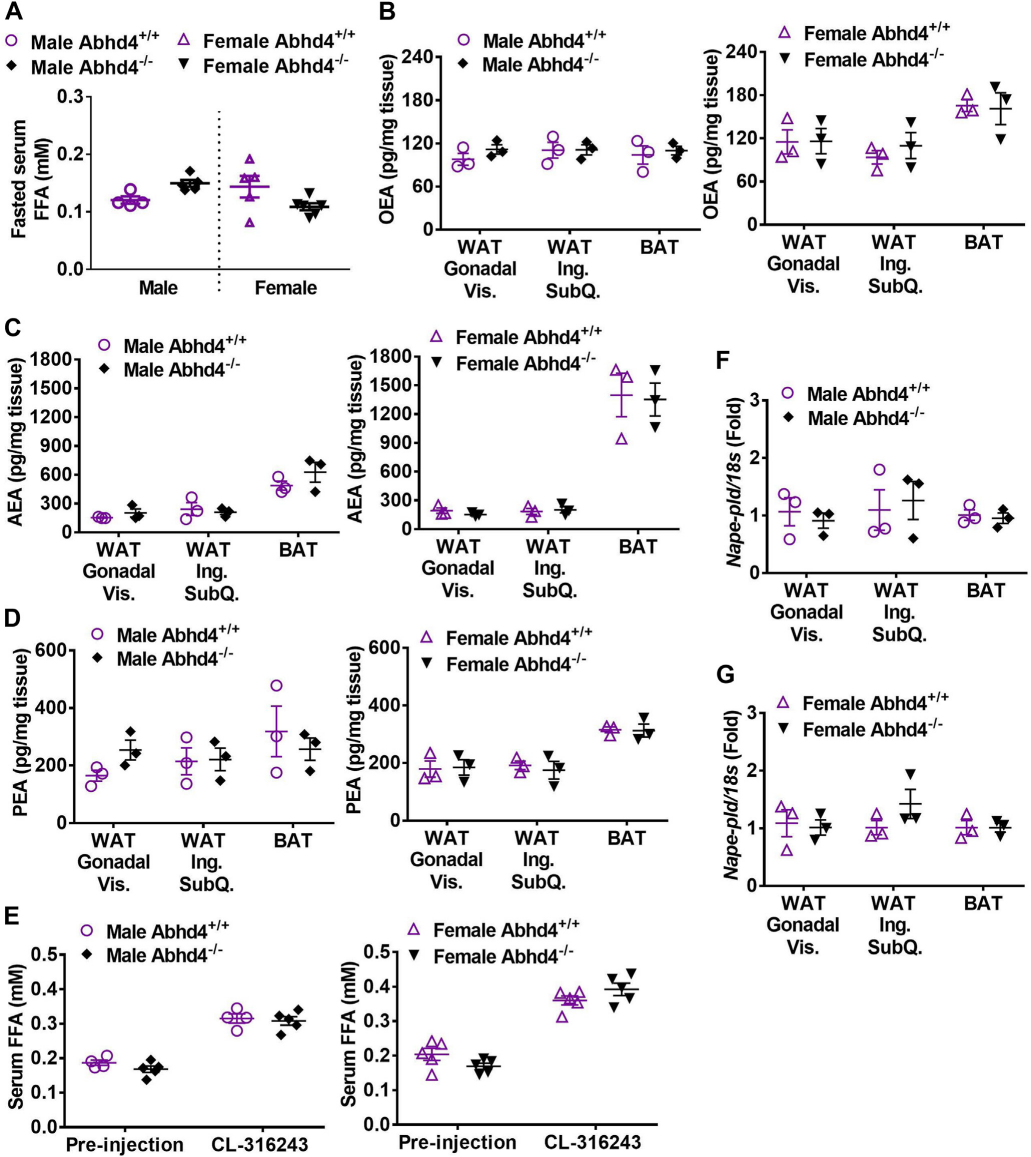
The effect of *Abhd4* deletion on lipolysis capacity and *N*-acyl ethanolamine production. After a 20-weeks of a high fat diet feeding, at 27 weeks of age, male and female whole body *Abhd4* knockout (*Abhd4*^−/−^) and their littermate control (*Abhd4*^+/+^) mice (n = 4–5/genotype/sex) were fasted for 16 h and then their serum, epididymal visceral white adipose tissue (WAT), and brown adipose tissue (BAT) samples were collected. A: Serum free fatty acid (FFA) concentrations were measured using an enzymatic colorimetric assay and adipose tissue (B) oleoylethanolamide (OEA), (C) arachidonoylethanolamide/anandamide (AEA), and (D) palmitoylethanolamide (PEA) levels were quantified using mass spectrometry. A–D: Results are analyzed using a two-tailed Student's unpaired *t* test. E: Seven weeks old male and female mice (n = 4–5/genotype) were on a high fat diet for 18 weeks and then injected intraperitoneally with CL-316,243 at 1 mg/kg body weight for 2 h. Mouse serum samples were collected, and FFA concentrations were measured using an enzymatic colorimetric assay. Results are analyzed using a two-way ANOVA with Sidak multiple comparisons. F–G: Tissue RNA was extracted and reverse-transcribed into cDNA for real-time PCR quantification of *Nape-pld* normalized to *18s* (endogenous control). Results are presented as the fold change compared to *Abhd4*^+/+^ and analyzed using a two-tailed Student's unpaired *t* test.

We then hypothesized that *Abhd4*^−/−^ and *Abhd4*^adipose−/−^ mice will have decreased adipose OEA content and lipolysis capacity during fasting. Surprisingly, we did not observe any difference in fasted serum free fatty acid concentrations (Fig. 8A) and adipose OEA levels (Fig. 8B) in both male and female *Abhd4*^−/−^ mice compared to *Abhd4*^+/+^ mice on a high-fat diet. In addition, we examined whether the deletion of *Abhd4* affects the production of other NAEs such as AEA and PEA. Similar to OEA results, fasted adipose AEA (Fig. 8C) and PEA (Fig. 8D) levels were not different between the genotypes (both male and female).

We also examined the effect of *Abhd4* deletion on lipolysis induced by CL-316,243, a potent β-adrenergic receptor (AR) agonist highly specific for rodent β3-AR (26, 42). We observed no difference in CL-316,243 induced lipolysis, measured by serum free fatty acid concentration, between *Abhd4*^+/+^ and *Abhd4*^−/−^ (both male and female) on a high-fat diet (Fig. 8E).

Additionally, as NAE can be converted from NAPE via the classical NAPE-PLD pathway or the alternative ABHD4 pathway (Fig. 1A), we investigated *Nape-pld* expression in the adipose tissue of *Abhd4*^−/−^ mice to determine if it could compensate for the loss of *Abhd4*. Deletion of *Abhd4* had no effect on the *Nape-pld* gene expression in both male (Fig. 8F) and female (Fig. 8G) mouse adipose tissue.

**Fig. 9:**
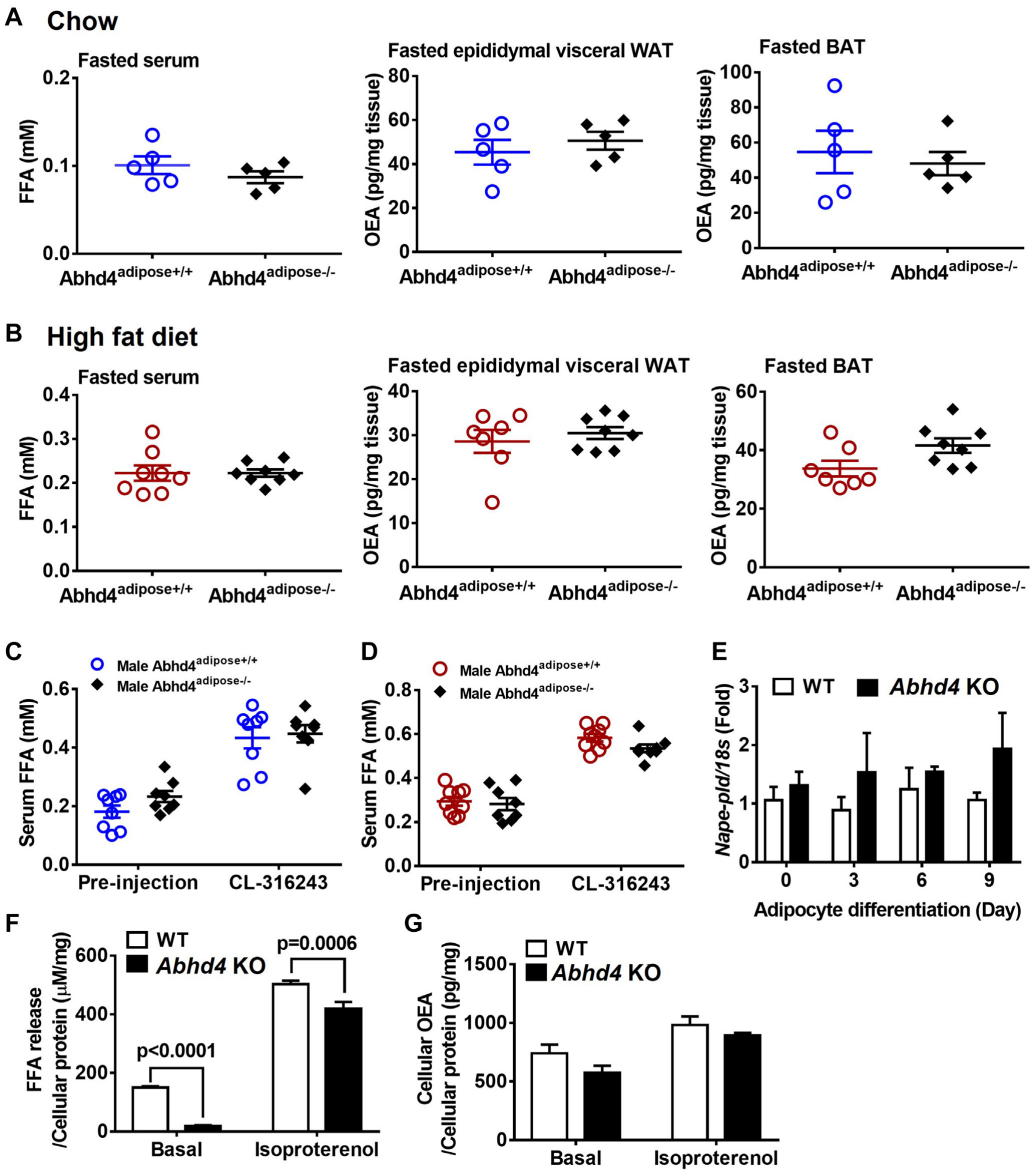
The effect of adipocyte *Abhd4* deletion on lipolysis capacity and oleoylethanolamide (OEA) production. A: After 16 h of fasting, serum, epididymal visceral white adipose tissue (WAT), and brown adipose tissue (BAT) samples were collected from chow-fed male adipocyte specific *Abhd4* knockout (*Abhd4*^adipose−/−^) and their littermate control (*Abhd4*^adipose+/+^) mice (n = 5/genotype) at the age of 22–24 weeks old. B: After a 16-weeks high fat diet feeding, at 24 weeks of age, male mice (n = 8/genotype) were fasted for 16 h and then euthanized to collect serum, epididymal visceral WAT, and BAT samples. Serum free fatty acid (FFA) concentrations were measured using an enzymatic colorimetric assay and adipose OEA levels were quantified using mass spectrometry. (A, B) Results are analyzed using a two-tailed Student's unpaired *t* test. Another cohort of (C) chow-fed (n = 8/genotype) and (D) a high fat diet-fed (n = 8–9/genotype) male mice were injected intraperitoneally with CL-316,243 at 1 mg/kg body weight for 2 h. Mouse serum samples were collected and FFA concentrations were measured using an enzymatic colorimetric assay. E: Cellular RNA was extracted from wildtype (WT) control and *Abhd4* knockout (KO) 3T3-L1 cells (n = 3/genotype) and reverse-transcribed into cDNA for real-time PCR quantification of *Nape-pld* nomalized to *18s* (endogenous control). Results are presented as the fold change compared to WT 3T3-L1 at Day 0. F, G: Day 9 differentiated WT control and *Abhd4* KO 3T3-L1 adipocytes were treated without (i.e., water) or with 10 μM isoproterenol for 3 h (n = 5/genotype/treatment). Culture media were collected for the measurement of FFA concentrations using an enzymatic colorimetric assay and cellular OEA levels were quantified using mass spectrometry. C–G: Results are analyzed using a two-way ANOVA with Sidak multiple comparisons.

Similarly, we did not observe any difference in fasted serum free fatty acid concentrations and adipose OEA levels in male *Abhd4*^adipose−/−^ mice compared to *Abhd4*^adipose+/+^ on chow (Fig. 9A) or fed a high-fat diet (Fig. 9B). Fasted adipose PEA and AEA levels were also not different between the genotypes on chow (supplemental Fig. S2A) or fed a high-fat diet (supplemental Fig. S2B). Furthermore, there was no difference in CL-316,243 induced lipolysis between male *Abhd4*^adipose+/+^ and *Abhd4*^adipose−/−^ on chow (Fig. 9C) or fed a high-fat diet (Fig. 9D). We observed similar results in female *Abhd4*^adipose−/−^ versus *Abhd4*^adipose+/+^ mice on chow or a high-fat diet (Data not shown).

Finally, we tested whether deletion of *Abhd4* affected OEA production and lipolysis in vitro since *Abhd4* deletion caused a strong phenotype (e.g., TAG accumulation) in 3T3-L1 cells (Fig. 2, Fig. 3, Fig. 4). It should be noted that the loss of *Abhd4* did not affect the expression of *Nape-pld* mRNA during 9 days of WT 3T3-L1 adipocyte differentiation (Fig. 9E). WT and ABHD4-deficient adipocytes (Day 9) were treated without (basal lipolysis) or with isoproterenol, a non-selective β-AR agonist, to stimulate lipolysis. Compared to WT adipocytes, ABHD4 deficient adipocytes had reduced basal and stimulated lipolysis, measured as attenuated free fatty acid release from cells into the medium (Fig. 9F). However, OEA levels did not change in ABHD4-deficient adipocytes versus control adipocytes at basal or isoproterenol-induced lipolysis (Fig. 9G).

## Discussion

ABHD4 is a NAPE/lyso-NAPE lipase (Fig. 1A). Human ABHD4 is a 342-residue protein (38.8 kDa) encoded by 8 exons located on chromosome 14q11.2. Mouse ABHD4 is a 337-residue protein with 99% amino acid identity with human ABHD4. The Cravatt lab reported that *Abhd4* is highly expressed in mouse brain (11). We used the GTEx RNAseq dataset to examine *ABHD4* mRNA profiles across all human tissues and found that adipose tissues demonstrate the second highest *ABHD4* mRNA expression (reproductive organs have the highest *ABHD4* mRNA expression) (https://gtexportal.org/home/gene/ABHD4#geneExpression). We further examined the relationship between adipose *ABHD4* expression and obesity. Our collaborators observed that *ABHD4* gene expression in human subcutaneous adipose tissue (located beneath the skin) is positively correlated with Body Mass Index (BMI, kg/m^2^) in the African American Genetics of Metabolism and Expression (AAGMEx) cohort (r = 0.13, *P* = 0.0000365), a cohort of 256 African Americans from North Carolina with in-depth glucometabolic phenotyping and adipose tissue transcriptome analysis (43). In addition to the human cohorts, adipose *Abhd4* expression positively correlates with retroperitoneal visceral (located in the abdominal cavity) fat pad weight (n = 430, r = 0.15, *P* = 0.002) in an outbred rat model (43). The human cohort, an outbred rat model, and our diet-induced obese mouse study (Fig. 1B–F) demonstrate for the first time that adipose ABHD4 gene expression is positively associated with obesity, suggesting a previously unappreciated role for ABHD4 in controlling adipose TAG homeostasis.

To better understand the role of ABHD4 in adiposity, we generated an *Abhd4* KO mouse 3T3-L1 pre-adipocyte using CRISPR gene editing. Our cell study shows for the first time that deletion of *Abhd4* in 3T3-L1 pre-adipocytes promotes adipogenesis and lipogenesis (Fig. 2, Fig. 3, Fig. 4) and suppresses lipolysis (Fig. 9F). The underlying mechanisms may go beyond simply catabolizing NAPE and synthesizing NAE as we observe no difference in NAE such as OEA production between ABHD4-deficient adipocytes and their WT control adipocytes (Fig. 9G). Similarly, we observe no difference in adipose tissue OEA, AEA, and PEA production (Figs. 8C, D, 9A, B, and S2) between *Abhd4* KO (both whole body and adipocyte-specific) and their littermate control mice (both male and female) on chow or a high-fat diet. Furthermore, the overall metabolic phenotypes (Figs. 5 and 6) are not different between *Abhd4* KO (both whole body and adipocyte-specific) and their littermate control mice (both male and female) on chow or a high-fat diet. Indeed, the Cravatt lab found that although GP-NAE (the intermediate) decreased, neither NAPE (the substrate) nor NAE (the product) was significantly affected in the brain of whole-body *Abhd4* KO mice (19). This may be due to the direct conversion from NAPE to NAE by NAPE-PLD which controls steady-state concentrations of these lipids. The compensation, however, was not caused by NAPE-PLD expression (Figs. 8F, G and 9E) but may have been caused by its activity (not yet determined).

How does deletion of *Abhd4* promote adipogenesis and lipogenesis if NAPE and NAE metabolism is not responsible for this cell phenotype. A recent study surveying protein-protein interactions across the adult mouse brain identified that ABHD4 is physically binding to the regulatory β subunits of protein kinase CK2 (previously called casein kinase 2 or CK-II), possibly regulating its protein stability and enzyme activity (44). Protein kinase CK2 is a ubiquitously expressed and constitutively active serine/threonine protein kinase (45). Structurally, mammalian protein kinase CK2 is a tetrameric enzyme, composed of two catalytic (α and α′) and two regulatory (β) subunits. The regulatory β subunit does not control the catalytic activity of the CK2α or CK2α′ subunits. CK2β regulates CK2’s protein stability (46), substrate specificity (47), and the ability to attach and penetrate the cell membrane (48). The constitutively active protein kinase CK2 phosphorylates hundreds of protein substrates and controls numerous cellular processes including adipocyte differentiation (45, 49).

Previous studies have demonstrated that protein kinase CK2’s protein expression and kinase activity was induced during the early stage of 3T3-L1 adipogenesis (< Day 6), but after Day 6, alteration of protein kinase CK2 expression and activity by its inhibitors have no effect on adipocyte maturation (50, 51, 52). In addition, the transcription factor C/EBPδ has been identified as a phosphorylated substrate for protein kinase CK2 to promote early adipogenesis by increasing *Pparγ* mRNA expression (53). Therefore, our future study will test a hypothesis that ABHD4 regulates the early stage of adipocyte differentiation by acting as a suppressor of protein kinase CK2. We have generated a 3T3-L1 cell line in which FLAG is knocked in to tag endogenous ABHD4 (as confirmed by immunoblotting, unpublished) for this future study. We propose that ABHD4 not only works as a lipase but can also physically interact with other proteins such as protein kinase CK2 in pre-adipocytes to retard adipocyte differentiation. Overall, in this study, we demonstrate that ABHD4 functions to regulate adipocyte differentiation in vitro, but ABHD4 is dispensable for adipose tissue expansion in high-fat diet-fed mice.

## Data Availability

The datasets that support the findings of this study are available in this article and the supplemental data.

## Supplemental data

This article contains supplemental data.

## Conflict of interests

The authors declare that they have no known competing financial interests or personal relationships that could have appeared to influence the work reported in this paper.
