# Supplementary figures and images for: ABHD4 regulates adipocyte differentiation in vitro but does not affect adipose tissue lipid metabolism in mice
Source: J Lipid Res. 2023 Jun 22;64(8):100405. doi: 10.1016/j.jlr.2023.100405 (PMC10400869; doi:10.1016/j.jlr.2023.100405)

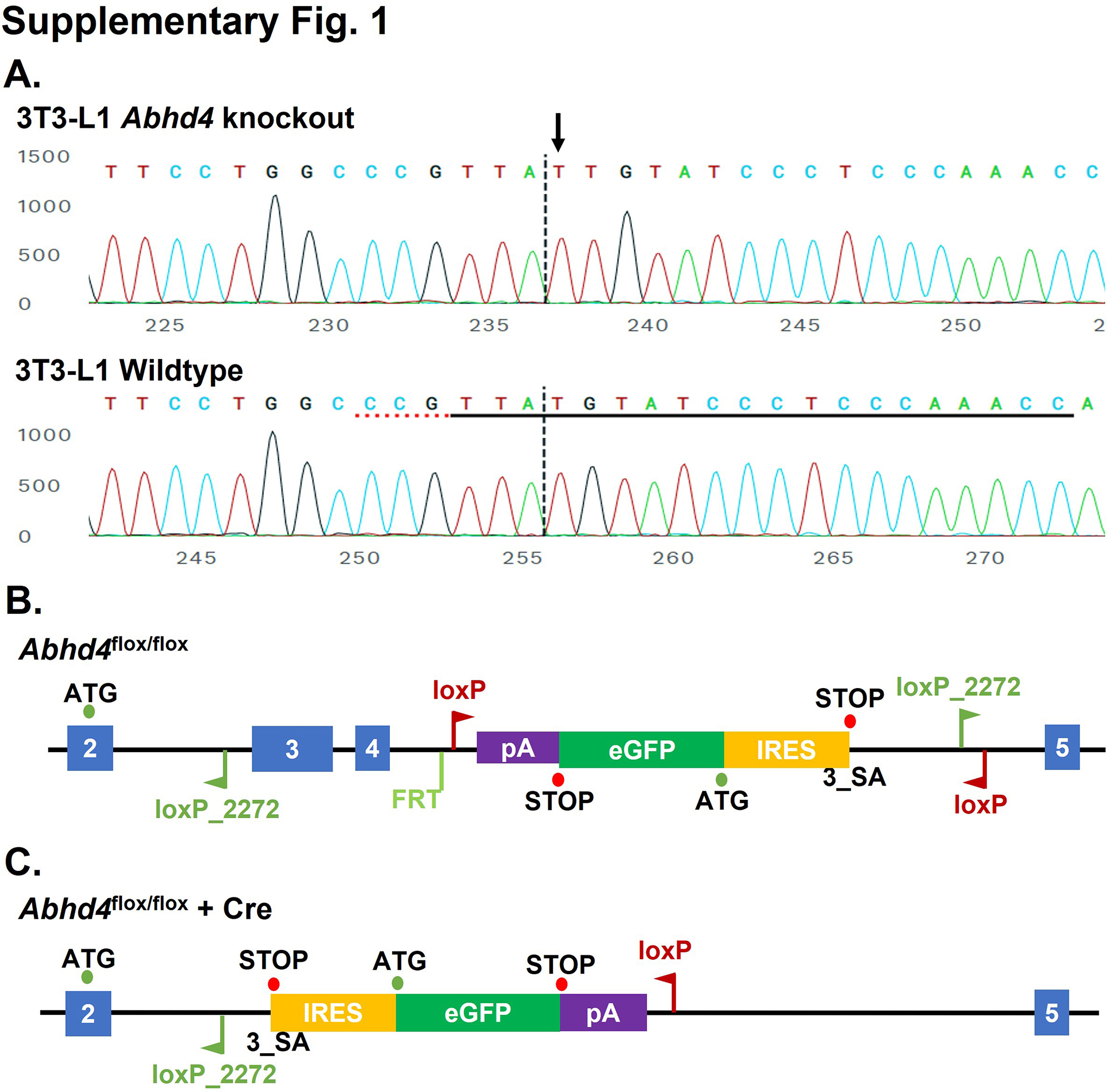

Supplement: Supplemental Fig 1 [file figs1.jpg]

# Supplementary Fig. 2

## A. Chow

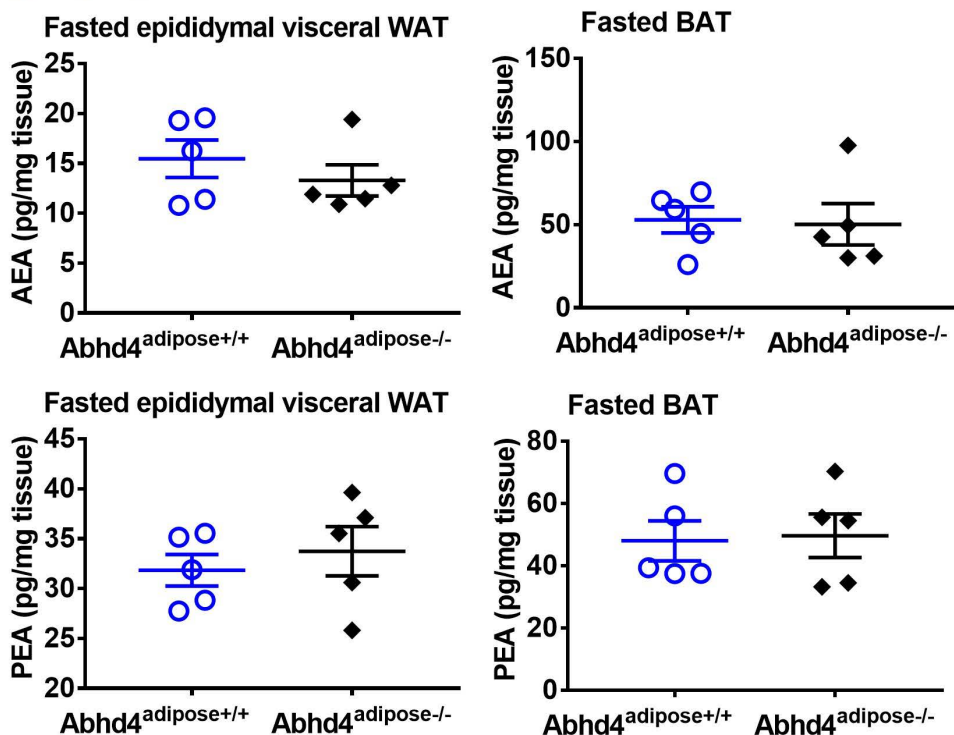

## B. High fat diet

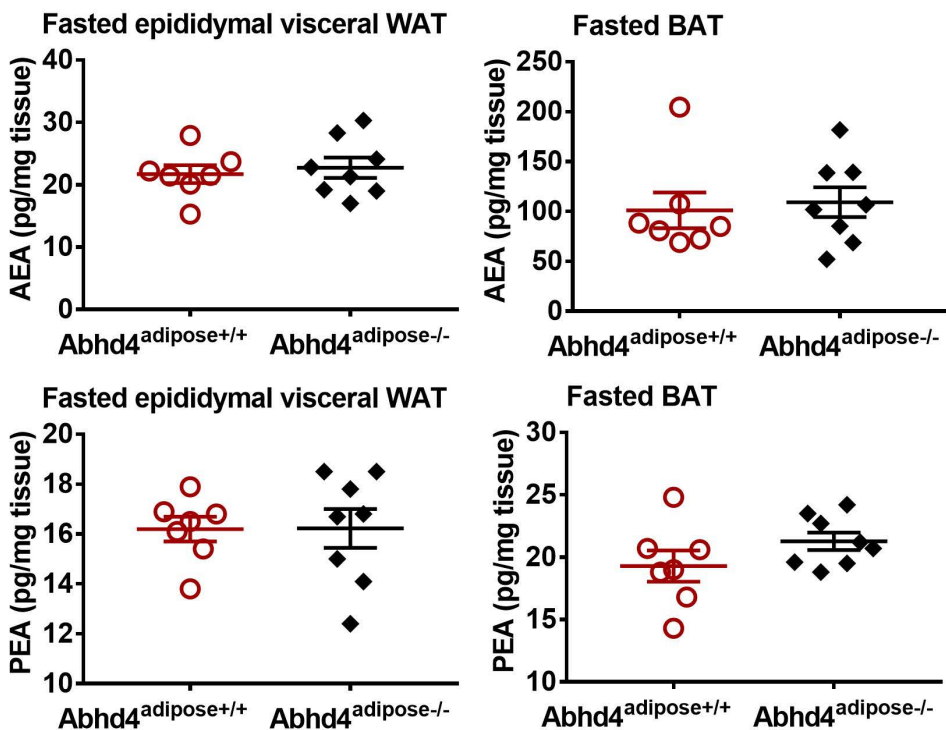

Supplement: Supplemental Fig 2 [file mmc1.pdf]
